# Опыт использования инсулиновой помпы TruCare III у пациентов с сахарным диабетом 1 типа

**DOI:** 10.14341/probl13516

**Published:** 2024-11-04

**Authors:** И. А. Барсуков, А. А. Демина

**Affiliations:** Московский областной научно-исследовательский институт им. М.Ф. Владимирского; Московский областной научно-исследовательский институт им. М.Ф. Владимирского

**Keywords:** инсулиновая помпа, сахарный диабет 1 типа, непрерывное мониторирование глюкозы

## Abstract

Возможности современной медицины существенно расширились с внедрением в практику устройств для постоянной подкожной инфузии инсулина (ППИИ, инсулиновых помп). Производство устройств для ППИИ расширяется с каждым годом, приводя к выходу на рынок инсулиновых помп различных по ценовой категории, но принципиально не различающихся в техническом плане. Появление новых моделей в Российской Федерации и, как следствие, расширение потенциального ассортимента устройств, может вызвать ряд практических вопросов как у пациента, так и у практикующего врача. В связи с этим крайне важным является оценка принципиально новых моделей, в том числе с позиции пользователя. Одним из таких устройств является инсулиновая помпа TruCare III (Уси Апекс Медикал К.; Лтд, Китай), получившая государственную регистрацию на территории Российской Федерации 15 сентября 2023 г. В настоящей статье представлен опыт использования данной модели инсулиновой помпы пациентами с сахарным диабетом 1 типа (СД1) с оценкой параметров гликемического контроля и описанием пользовательских нюансов.

## Abstract (en)

The development of devices for continuous subcutaneous insulin infusion (CSII or insulin pumps) dramatically improved medical care for patients with diabetes mellitus. Insulin pump production widens annually resulting in number of new models entering the market, that differs in price but are similar in technical features. The entering of such new models to the Russian market can cause practical issues both in patient and in health care provider, so their estimation is of great importance. Insulin pump TruCare III (Apex Medical Co., Ltd, China) was registered in Russian Federation in September 15, 2023. The clinical experience of the TruCare III use in patients with type 1 diabetes mellitus is presented with the focus on glycemic parameters and custom features.

## АКТУАЛЬНОСТЬ

Достижение целевых показателей гликемического контроля является важнейшим компонентом лечения пациентов с сахарным диабетом (СД) [1]. Возможности современной медицины существенно расширились с внедрением в практику устройств для постоянной подкожной инфузии инсулина (ППИИ, инсулиновых помп) [2][3]. Согласно имеющимся данным, количество пациентов с СД 1 типа (СД1), использующих инсулиновую помпу, увеличивается с каждым годом. По данным регистра больных с СД на 2021 г., около 66% пациентов с СД1 использовали инсулиновую помпу в США [4]. В Китае частота использования помповой инсулинотерапии для контроля СД1 составляет 11,4% [5]. По данным Федерального регистра сахарного диабета на 2023 г., количество взрослых пациентов в Московской области, постоянно использующих инсулиновую помпу, составляет 915 человек.

Преимущества помповой инсулинотерапии перед режимом множественных инъекций инсулина (МИИ) в улучшении показателей гликемии и качества жизни были неоднократно доказаны в ходе крупных рандомизированных клинических исследований и метаанализов [6-8]. При этом возможность незамедлительно регулировать дозу вводимого инсулина особенно актуальна, так как позволяет предотвратить развитие гипогликемии, в том числе во время физической нагрузки [9].

Производство устройств для ППИИ расширяется с каждым годом, приводя к выходу на рынок инсулиновых помп различных по ценовой категории, но принципиально не различающихся в техническом плане [10]. Широкое распространение рынке имеют инсулиновые помпы компании Medtronic различных моделей и их аналоги (помпы TruCare III), а также беспроводные инсулиновые патч-помпы (V-GO (Mannkind, USA), Omnipod (Insulet Corp., USA)). Появление новых моделей в Российской Федерации и, как следствие, расширение потенциального ассортимента устройств может вызвать ряд практических вопросов как у пациента, так и у практикующего врача. В связи с этим крайне важным является оценка новых моделей, в том числе с позиции пользователя.

Одним из таких устройств является инсулиновая помпа TruCare III (Уси Апекс Медикал К.; Лтд, Китай), получившая государственную регистрацию на территории Российской Федерации 15 сентября 2023 г. По своим техническим характеристикам, назначению, сфере применения, функциональным возможностям, конструктивному исполнению, принципу работы, а также степени безопасности данное устройство эквивалентно инсулиновой помпе MiniMed Paradigm MMT-715 (Medtronic Minimed, USA), ранее зарегистрированной на территории Российской Федерации и активно использовавшейся пациентами с СД. Как следствие, в соответствии с пунктом 37 Приказа МЗ РФ от 30 августа 2021 г. №885н «Об утверждении Порядка проведения оценки соответствия медицинских изделий в форме технических испытаний, клинических испытаний в целях государственной регистрации медицинских изделий» клинические испытания устройства проводились без участия человека в форме оценки и анализа клинических данных и сравнения технических характеристик с ранее зарегистрированным аналогом (табл. 1). Выводы по сравнительному анализу свидетельствуют об эквивалентности TruCare III зарегистрированному аналогу и обуславливают возможность принятия решения о взаимозаменяемости в соответствии с установленной процедурой.

В настоящей статье представлен опыт использования данной модели инсулиновой помпы пациентами с СД1 с оценкой параметров гликемического контроля и описанием пользовательских нюансов.

**Table:** Таблица 1. Сравнение инсулиновой помпы TruCare III с эквивалентным медицинским изделием MiniMed Paradigm ММТ- 715

Наименование показателя, ед.изм. показателя	MiniMed Paradigm, модель ММТ-715	TruCare III
Интерфейс управления	Интеллектуальное русифицированное меню	Интеллектуальное русифицированное меню
Размеры, мм (длина*ширина*глубина)	94*51*20	88*58*20
Диапазон базальной дозы, Ед/час	0,025–35	0,025–35
Временная базальная скорость, %	0–200	0–200
Шаг программирования временной базальной дозы, %	1	1
Временная базальная скорость (временной интервал), минимальный, мин	30	30
Максимальная болюсная доза инсулина, на 1 болюс, Ед/час	25	25
Шаг изменения базальной дозы, Ед/час	0,025	0,025
Встроенный калькулятор расчета болюсной дозы инсулина с учетом активного инсулина	Наличие	Наличие
Типы подачи сигнала тревоги	звук, вибрация	звук, вибрация
Водонепроницаемость по стандарту IEC60529	IPX8	IPX8
Состояние одиночной неисправности вызывает остановку введения инсулина помпой. Максимальная инфузия при состоянии одиночной неисправности составляет, ед.	0,2	0,2

## ОПИСАНИЕ СЛУЧАЯ

Пять пациентов с СД1, ранее использовавшие инсулиновые помпы Medtronic MiniMed 720G или Medtronic MiniMed 722 (Medtronic Minimed, USA), были переведены на TruCare III (Уси Апекс Медикал К.; Лтд, Китай).

Каждому пациенту, подписавшему добровольное информированное согласие, была установлена система непрерывного мониторирования глюкозы (НМГ) Freestyle Libre (Abbott Diabetes Care, Alameda, USA) и предложено подключение к платформе LibreView ГБУЗ МО МОНИКИ им. М.Ф. Владимирского для получения возможности удаленного наблюдения за динамикой уровня глюкозы врачом-исследователем и принятия решения о необходимости коррекции терапии.

Через две недели сбора данных о состоянии углеводного обмена системой НМГ пациентам проведена замена ранее использовавшейся инсулиновой помпы на модель TruCare III (Уси Апекс Медикал К.; Лтд, Китай), настроенной по тем же параметрам (в частности, скорости подачи инсулина в базальном режиме, настройкам помощника болюса и пр.).

Через две недели использования инсулиновой помпы TruCare III проведена повторная оценка параметров амбулаторного гликемического профиля (AGP) с последующим сравнительным анализом данных по следующим параметрам:

-среднее число сканирований/просмотров;

-время нахождения в целевом диапазоне (3,9–10,0 ммоль/л);

-время нахождения в диапазоне выше и ниже целевого значения;

-средний уровень глюкозы;

-вариабельность уровня глюкозы;

-количество гипогликемических явлений;

-средняя длительность гипогликемических явлений.

Помимо оценки параметров гликемического контроля, был проведен опрос пациентов в открытой форме о преимуществах и недостатках исследуемой модели с точки зрения пользователя. Основные характеристики пациентов представлены в таблице 2.

## Результаты физикального, лабораторного и инструментального исследования

Все пациенты проходили неоднократное обучение в школе сахарного диабета и были хорошо ориентированы в принципах работы инсулиновой помпы, что в том числе подтверждается достижением целевого показателя гликированного гемоглобина (табл. 2). Исходные параметры амбулаторного гликемического профиля (AGP) представлены в таблице 3. С клинической точки зрения пациенты практически не различались по времени нахождения датчика в активном состоянии, времени в целевом диапазоне (time in range, TIR), времени выше целевого диапазона (time above range, TAR), времени ниже целевого диапазона (time below range, TBR), коэффициенту вариации (coefficient of variation, CV).

**Table:** Таблица 2. Основные клинические параметры пациентов с сахарным диабетом 1 типа, переведенных на инсулиновую помпу TruCare III Примечание. ИМТ — индекс массы тела; HbA1c — гликированный гемоглобин.

Параметр/пациент	Пациент А	Пациент Б	Пациент В	Пациент Г	Пациент Д
Пол	мужской	женский	мужской	женский	женский
Возраст, лет		22	31	20	41
Длительность заболевания, лет	20	11	28	10	25
ИМТ, кг/м2	23,84	18,66	24,84	22,76	24,44
Уровень HbA1c, %	5,5	5,7	6,8	5,65	5,6
Суточная доза инсулина, Ед	55	20,4	110	53	95
Модель инсулиновой помпы	Medtronic MiniMed 720G	Medtronic MiniMed 720G	Medtronic MiniMed 720G	Medtronic MiniMed 722	Medtronic MiniMed 720G

**Table:** Таблица 3. Исходные параметры амбулаторного гликемического профиля пациентов (%) Примечание. TIR — время в целевом диапазоне; TBR (Уровень 1) — время ниже целевого диапазона <3,9 ммоль/л; TBR (Уровень 2) — время ниже целевого диапазона <3,0 ммоль/л; TAR (Уровень 1) — время выше целевого диапазона >10,0 ммоль/л; TAR (Уровень 2) — время выше целевого диапазона > 13,9 ммоль/л; CV — коэффициент вариации.

Пациент/параметр	Доля времени с активным устройством	TIR	TBR (Уровень 1)	TBR (Уровень 2)	TAR (Уровень 1)	TAR (Уровень 2)	CV
Пациент А	95	86	8	1	4	1	33,5
Пациент Б	90	95	3	0	2	0	24,6
Пациент В	94	93	4	1	2	0	25,6
Пациент Г	98	86	4	0	9	1	32,7
Пациент Д	100	88	1	0	11	0	26,6

## Исход и результаты последующего наблюдения

Через 2 недели пациентам была установлена инсулиновая помпа TruCare III (Уси Апекс Медикал К.; Лтд, Китай) с последующей оценкой AGP (таблица 4). При проведении сравнительного анализа не было выявлено существенных клинических отличий в оцениваемых параметрах.

Так, у пациента А на фоне использования изучаемой инсулиновой помпы отмечалось некоторое улучшение параметров гликемического контроля: увеличение TIR с 86 до 96%, снижение TBR 1 уровня с 8 до 2% и отсутствие TBR 2 уровня (рис. 1).

Схожая картина была получена при анализе AGP пациента Б: при неизменном времени нахождения в целевом диапазоне отмечено снижение времени ниже целевого диапазона 1 уровня (рис. 2).

В то же время у пациента Г было отмечено увеличение времени ниже целевого диапазона 1 уровня с 4 до 8% и коэффициента вариации с 32,7 до 38,9% с одновременным снижением времени выше целевого диапазона 1 уровня с 9 до 4%. При этом TIR существенно не изменилось (табл. 2 и 3). Подобное обстоятельство обусловлено тем, что в исследуемый период пациенту проводилась коррекция терапии (в частности, увеличение скорости подачи инсулина в базальном режиме и коррекция углеводного коэффициента) с целью улучшения параметров гликемического контроля. Увеличение частоты легких гипогликемических реакций на фоне увеличения дозы инсулина внесло дополнительный вклад в увеличение коэффициента вариации, что в дальнейшем потребовало проведения дополнительной работы с пациентом по коррекции лечения. С практической точки зрения описываемые изменения параметров гликемии отражают рутинную клиническую работу с пациентом по динамическому наблюдению и лечению и не зависят от используемой модели инсулиновой помпы с учетом одинаковых технических характеристик (табл. 1).

На фоне использования инсулиновой помпы TruCare III пациенты отмечали следующие нюансы:

-удобство крышки отсека для батареи/аккумулятора. В отличие от моделей Medtronic форма крышки в виде шестеренки позволяет откручивать ее как руками, так и с помощью ключа. Аналогичная технология используется в помпах Animas Onetouch Ping;

-в том случае, когда в резервуаре инсулиновой помпы остается менее 51,2 Ед, заправка инфузионной системы и дальнейшее использование помпы невозможны. При этом помпа выдает ошибку. Подобный подход, с одной стороны, используется из соображений безопасности, с другой — требует дополнительного информирования пациента во избежание осложнений;

-в случае установки батареи с неполным зарядом помпа выдает ошибку с требованием ее заменить. Как и в предыдущем случае, основной целью является обеспечение безопасности пациента, в частности избежание неожиданного выключения инсулиновой помпы.

В целом пациенты позитивно оценили использование инсулиновой помпы TruCare III, что было продемонстрировано опросами в открытой форме и данными опросника удовлетворенности лечением СД (DTSQ) (табл. 5).

**Table:** Таблица 4. Параметры амбулаторного гликемического профиля пациентов на фоне использования инсулиновой помпы TruCare III в течение 14 дней (%) Примечание. TIR — время в целевом диапазоне; TBR (Уровень 1) — время ниже целевого диапазона <3,9 ммоль/л; TBR (Уровень 2) — время ниже целевого диапазона <3,0 ммоль/л; TAR (Уровень 1) — время выше целевого диапазона >10,0 ммоль/л; TAR (Уровень 2) — время выше целевого диапазона >13,9 ммоль/л; CV — коэффициент вариации.

Пациент/параметр	Доля времени с активным устройством	TIR	TBR (Уровень 1)	TBR (Уровень 2)	TAR (Уровень 1)	TAR (Уровень 2)	CV
Пациент А	95	96	2	0	2	0	23,2
Пациент Б	89	95	1	0	4	0	23,7
Пациент В	100	95	5	0	0	0	22,4
Пациент Г	100	85	8	1	4	2	38,9
Пациент Д	100	87	1	0	10	2	31,1

**Figure:**
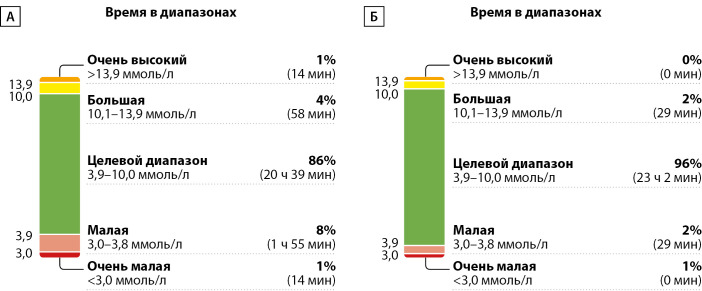
Рисунок 1. Динамика амбулаторного гликемического профиля у пациента А исходно (А) и через 2 недели использования инсулиновой помпы TruCare III (Б).

**Figure:**
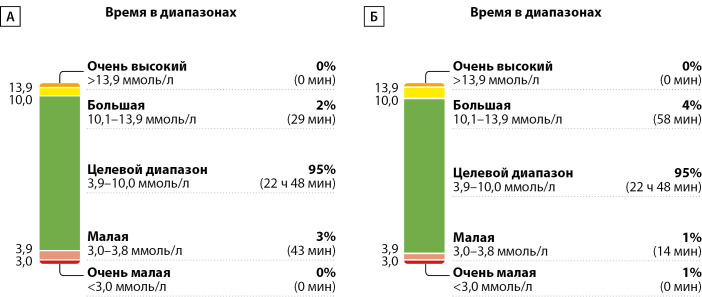
Рисунок 2. Динамика амбулаторного гликемического профиля у пациента Б исходно (А) и через 2 недели использования инсулиновой помпы TruCare III (Б)

**Table:** Таблица 5. Оценка удовлетворенности лечением диабета у пациентов исходно и на фоне использования инсулиновой помпы TruCare III по опроснику DTSQs

Пациент/параметр	Исходно	На фоне использования TruCare III
Пациент А	39	40
Пациент Б	37	37
Пациент В	44	42
Пациент Г	35	28
Пациент Д	34	33

## ОБСУЖДЕНИЕ

Расширение ассортимента моделей инсулиновых помп, в том числе с различной ценовой категорией расходных материалов, позволяет повысить доступность подобного рода устройств для пациентов с СД. Информирование практикующего врача о преимуществах и недостатках новых моделей имеет первостепенное значение для повышения качества оказания медицинской помощи.

В представленной работе продемонстрирован опыт использования инсулиновой помпы TruCare III (Уси Апекс Медикал К.; Лтд, Китай) пациентами с СД1, а также описаны пользовательские нюансы, способствующие комфортному использованию устройства. Отметим, что в рамках настоящей работы целенаправленно были отобраны пациенты с исходно хорошей компенсацией углеводного обмена и длительным стажем заболевания, которые неоднократно проходили обучение в школе сахарного диабета и обладают достаточным уровнем знаний как о заболевании, так и о тонкостях использования инсулиновой помпы. Авторы не исключают, что пациентам, не достигшим целевых показателей углеводного обмена на фоне использования инсулиновой помпы иной модели, в случае принятия решения о переходе на помпу TruCare III может потребоваться дополнительное обучение в школе СД с фокусом на помповую инсулинотерапию или же перевод на новую модель в условиях стационара.

## Заключение

Инсулиновая помпа модели TruCare III (Уси Апекс Медикал К.; Лтд, Китай) не отличается по техническим характеристикам от иных моделей и соответствует государственным стандартам.

На фоне использования инсулиновой помпы TruCare III (Уси Апекс Медикал К.; Лтд, Китай) у большинства пациентов не было выявлено клинически значимого ухудшения параметров гликемического контроля, что было продемонстрировано на основании данных амбулаторного гликемического профиля.

Использование инсулиновой помпы модели TruCare III (Уси Апекс Медикал К.; Лтд, Китай) может быть рекомендовано пациентам с СД в качестве альтернативы иным моделям, представленным на рынке, при условии достаточного уровня знаний у пациента о своем заболевании и принципах работы с инсулиновой помпой.

Пациенты, решившие начать использование инсулиновой помпы TruCare III (Уси Апекс Медикал К.; Лтд, Китай), должны быть информированы лечащим врачом об особенностях работы данного устройства, в частности о лимите остаточного инсулина в резервуаре для заправки инфузионной системы (объем инсулина должен быть более 51,2 Ед), необходимости установки только новой батареи/аккумулятора в инсулиновую помпу.

## Дополнительная информация

Источники финансирования. Работа выполнена при финансовой поддержке компании «Эрвин» в соответствии с Договором на проведение научно-исследовательской работы с ГБУЗ МО МОНИКИ им. М.Ф. Владимирского № 007/2024-Н от 29.05.2024

Конфликт интересов. Авторы декларируют отсутствие явных и потенциальных конфликтов интересов, связанных с содержанием настоящей статьи.

Участие авторов. Барсуков И.А. — разработка концепции и дизайна исследования, внесение в рукопись существенной правки с целью повышения научной ценности статьи; Демина А.А. — получение, анализ данных и интерпретация результатов, написание статьи.

Все авторы одобрили финальную версию статьи перед публикацией, выразили согласие нести ответственность за все аспекты работы, подразумевающую надлежащее изучение и решение вопросов, связанных с точностью или добросовестностью любой части работы.

Согласие пациента. Все пациенты добровольно подписали информированные согласия на публикацию персональной медицинской информации в обезличенной форме.
